# Posttranslational Modifications of Rev-Erb*α* Protein and Abnormal Inflammatory Response in Gastric Cancer

**DOI:** 10.1155/2022/6291656

**Published:** 2022-12-28

**Authors:** Chen Ke, Cheng Xiaowen, Wan Yufeng, Li Dongchang, Zhu Huaqing, Wang Zhengguang

**Affiliations:** ^1^Department of General Surgery, The First Affiliated Hospital of Anhui Medical University, Hefei, Anhui, China; ^2^Department of Vascular Surgery, The Drum Tower Hospital Affiliated to Medical School of Nanjing University, Nanjing, Jiangsu, China; ^3^Department of Clinical Laboratory, The First Affiliated Hospital of Anhui Medical University, Hefei, Anhui, China; ^4^Laboratory of Molecular Biology and Department of Biochemistry, Anhui Medical University, Hefei, Anhui, China; ^5^Department of Otolaryngology, The Affiliated Chaohu Hospital of Anhui Medical University, Hefei, Anhui, China

## Abstract

We reported that Rev-erb*α*, a transcriptional repressor, is reduced in human gastric cancer and that it inhibits glycolysis in cultured gastric cancer cells. However, it is unclear whether Rev-erb*α* undergoes posttranslational modifications in gastric cancer. Here, we determined levels of Rev-erb*α* and its posttranslational modifications including phosphorylation, SUMOylation, and ubiquitination in N-methyl-N-nitrosourea (MNU)/*Helicobacter pylori* (*H. pylori*)-induced gastric cancer in mice and in cultured human gastric cancer cells. Administration of MNU plus *H. pylori* infection successfully induced gastric tumor in C57BL/6J mice. MNU/*H. pylori* decreased the levels of Rev-erb*α* in gastric tumor tissues of mice accompanied by an increase in the level of lactic acid. Rev-erb*α* protein SUMOylation and ubiquitination modifications were significantly increased, whereas phosphorylation was unchanged, in gastric cancer cells line BGC-823 and MNU/*H. pylori*-induced mouse gastric cancer tissues. Using human gastric cancer tissues, we found that Rev-erb*α* was specifically reduced in mucosal epithelial cells in gastric tissue. Cytokine levels were increased in MNU/*H. pylori*-exposed mice compared with control mice. Similarly, the levels of IL-6 IL-10, TNF-*α*, and VEGF were higher in the BGC-823 cell line compared with GES-1 cells. IL-6 and IL-1 incubation did not affect Rev-erb*α* levels in BGC-823 cells. Furthermore, Rev-erb*α* was recruited on the promoters of these cytokine genes, which suppressed their expression. Conclusively, Rev-erb*α* SUMOylation and subsequent ubiquitination may contribute to its protein reduction, which leads to increased glycolysis and abnormal inflammatory responses during the development of gastric cancer. Targeting Rev-erb*α* and its SUMOylation represents promising approaches for prevention and management of gastric cancer.

## 1. Introduction

Although gastric cancer is a common malignant tumor in humans, its pathological mechanism is not fully understood. The occurrence of gastric cancer is considered a result of multiple biological, genetic, and environmental factors, and multiple stages. *Helicobacter pylori* (*H. pylori*) infection is the most important factor. After its infection, *H. pylori* causes an inflammatory response in the gastric mucosa, which induces the host to produce a variety of cytokines that alter the microenvironment including the physiology and immune status of the stomach. This can lead to cancerous transformation and the unlimited growth of gastric mucosal epithelial cells [1–3]. With the development of metabolomics, recent research has advanced our understanding of the relationship between metabolic regulation and cancer. Extensive research has demonstrated that metabolic reprogramming is a hallmark of cancer and is intricately linked to oncogenesis and cancer immune escape. The concentration of lactic acid is consistently increased in the urine and/or tissue samples of gastric cancer patients, whereas glucose is considerably depleted compared with healthy individuals. These high lactate levels might be attributed to the special metabolism of most cancer cells because tumor cells consume a large amount of glucose for glycolysis even under the condition of sufficient oxygen (Warburg effect) [4]. This glycolytic switch was reported to be associated with oncogenic transformation and molecular signal transduction [5].

Rev-erb*α* is a nuclear receptor and critical component of the molecular clock that drives the daily rhythms of metabolism. Rev-erb family members participate in pathological processes, including sleep disorders, diabetes, atherosclerosis, Alzheimer's disease, and other diseases, by regulating the biological clock, inflammatory/immune responses, and lipid metabolism. A study revealed that Rev-erb*α* KO mice had a greater inflammatory response to cigarette smoke, including increased neutrophil lung influx and proinflammatory cytokine release compared with wildtype mice [6]. Stimulation of Rev-erb*α* activity by SR9009 greatly diminished ventilator-induced lung injury, inflammatory cell infiltration, and the production of the proinflammatory cytokine TNF-*α* [7]. Rev-erb*α* is critical for the regulation of inflammation- and metabolism-related gene transcription. We have examined the relationship between Rev-erb*α* and tumors [8–13]. Inflammation is usually related to the occurrence and development of cancer. The inducement of chronic inflammation increases the risk of cancer or promotes cancer progression, including *H. pylori* infection [14]. Inflammatory cytokines IL-6 is highly upregulated in many cancers and is considered to be one of the most important cytokine families in tumorigenesis and metastasis [15]. Inflammatory cytokines TNF-*α* can trigger the first step of tumor transformation, act as an autocrine growth factor for tumor cells, and play a major role in metastasis [16]. Previous studies showed that the decreased activity of Rev-erb*α* or Rev-erb*α* knockout promoted the production of TNF-*α* and IL-6 in rodent lungs [6, 7]. Knockdown of Rev-erb*α* is effective at modulating the production of IL-6 [17]. The Rev-erb*α* agonist SR9011 stimulated the expression of the anti-inflammatory cytokine IL-10 [18]. Upregulation of VEGF expression during gastric inflammation may be related to the development of gastric cancer [19]. We previously reported that Rev-erb*α* is reduced in human gastric cancer [13] and that it inhibits glycolysis in cultured gastric cancer cells [20]. Rev-erb*α* can undergo various protein modifications including phosphorylation which affects its stability. The current study investigated whether Rev-erb*α* reduction is associated with its posttranslational modifications, including phosphorylation, SUMOylation, and ubiquitination in gastric cancer. Inflammation and lactate levels were also measured during the development of gastric cancer.

## 2. Materials and Methods

### 2.1. Patients and Samples

Six fresh gastric cancer tissue pairs (tumor and adjacent normal tissues) were obtained by surgical resection at a similar time to avoid circadian changes from the First Affiliated Hospital of Anhui Medical University and immediately stored at ‒ 80°C. Tumor-adjacent tissues were obtained from as far away as 2 cm from the gastric tumor. All cases were diagnosed by histopathology. The tissue wax blocks and sections of gastric cancer were obtained from the Department of Pathology of the First Affiliated Hospital of Anhui Medical University and confirmed via histopathological diagnosis by a pathologist. Characteristics are shown in Table 1. Informed consent was obtained from each enrolled patient.

### 2.2. Animals

C57BL/6J mice (both males and females) used in the present research were purchased from the Model Animal Research Center of Nanjing University (Nanjing, China). In total, 60 4-week-old male C57BL/6J mice were housed in cages with a 12/12-hour light/dark cycle and maintained at 23°C under specific pathogen-free (SPF) conditions. Mice were divided into the following three groups: Group 1, control (*n* = 10). Group 2, N-methylnitrosourea (MNU) (Sigma Chemical Co., St. Louis, MO, USA) + *H. pylori* (ATCC, Manassas, VA, 6 months, *n* = 25). Group 3, MNU + *H. pylori* (12 months, *n* = 25). MNU was dissolved in distilled water at a concentration with 200 ppm and placed in a light-shielding bottle as drinking water for mice. Mice in the MNU groups were fed drinking water containing 200 ppm MNU twice a week for 10 weeks. After completion of MNU treatment, mice in the MNU + *H. pylori* groups were inoculated orogastrically with 5 × 10^9^colony-forming units/mL of *H. pylori* (ATCC 49179), five times every alternate day. Mice were sacrificed by CO_2_ asphyxiation at 6 and 12 months after inoculation at similar times to avoid circadian changes. All the experiments performed in this study were approved by the Ethics Committee of The First Affiliated Hospital of Anhui Medical University (Hefei, Anhui, China).

### 2.3. Cell Culture

Human gastric mucosal epithelial cells (GES-1) and human gastric cancer cell lines (BGC-823 and SGC-7901) were obtained from the American Type Culture Collection (Manassas, VA, USA). The cells were cultured in DMEM (Thermo Fisher Scientific, Beijing, China) with 10% fetal bovine serum and 100 IU/ml penicillin/streptomycin (Invitrogen, Carlsbad, CA, USA) at 37°C under 5% CO_2_ and 21% O_2_ condition.

### 2.4. Western Blot

Cell pellets or mouse gastric tissues were washed with phosphate-buffered saline (PBS) and homogenized with an appropriate amount of RIPA lysis buffer containing protease inhibitor cocktail and PMSF. The processed tissues were incubated in ice for 30 min. The supernatants were used as sample for experiment after centrifugation at 4°C with 12,000 ×g for 20 minutes. The BCA method was used to measure the protein concentration. Samples were separated by 10% SDS-PAGE and transferred to the PVDF membrane. The membranes were blocked with 5% fat-free milk in PBS containing 0.1% Tween 20 for 2 hours at room temperature, followed by overnight incubation with the primary antibody (Anti-Rev-erb*α* antibody: Santa Cruz, #sc-393215, 1 : 1,000) at 4°C. Next, the secondary antibody was incubated for 1 hour at room temperature, and the protein bands were detected using the ECL reaction solution.

### 2.5. Measurement of Lactate Concentrations

The cells as well as gastric tumor tissues of the MNU/*H. pylori* treatment groups and the corresponding tissues in the normal groups were collected under an empty stomach condition for the measurement of lactate concentrations using a lactate assay kit (BioVision, Milpitas, CA) in accordance with the manufacturer's instructions. Serum lactate was also measured in human subjects.

### 2.6. Hematoxylin and Eosin Staining

Gastric tissues were fixed them with 4% neutral buffered paraformaldehyde. These fixed lungs were embedded in paraffin, sectioned into 5 *μ*m sections using a rotary microtome, and stained with hematoxylin and eosin.

### 2.7. Immunofluorescence

Formalin-fixed paraffin-embedded tissues were cut int 4 *μ*m sections which were deparaffinized with xylene and rehydrated through a graded series of alcohols. The tissue sections were placed in a repair box filled with citric acid antigen retrieval solution, and antigen retrieval was performed in a microwave oven. The slides were washed 3 times with PBS after natural cooling, 5 min each time and then, blocking was done with 3% BSA in PBS buffer for 30 min. Tissues were incubated with primary antibodies against Rev-erb*α* (ab174309, Abcam) and Tff1 (ab92377, Abcam) at 1 : 200 dilutions overnight at 4°C in humidified chambers followed by incubation with the corresponding secondary antibody for 60 min at room temperature in the dark. The slides were mounted with resin after dropping an appropriate amount of an antifluorescence quencher on the tissue. Fluorescence was detected with fluorescence microscopy. Fluorescence was detected by using a LEICA TCS NT laser confocal microscope.

### 2.8. Immunoprecipitation (IP)

Cells and experimental gastric cancer tissues were lysed in IP lysis buffer containing 50 mM HEPES (pH7.4), 100 mM NaCl, 5 mM MgCl_2_, 0.5% NP-40, 10% glycerol, 1 mM NaF, 1 mM Na_3_VO_4_, and 1 × protease inhibitor cocktail for 20 min in ice. The cell lysate was centrifuged at 13,000 ×g at 4°C for 20 min. An appropriate amount of supernatant was taken for determination of protein concentration and prepared for input samples. Lysate containing about 400 *μ*g–2 mg of total protein and equal volume of precooled immunoprecipitation buffer containing an appropriate proportion of protease inhibitors and antibody-coupled agarose beads were added, respectively. The abovementioned mixture was incubated for overnight at 4°C. The precipitated complex was collected by centrifugation at 3000 rpm for 1 min and then washed with TBST three times. The immune complex was dissociated from the beads for Western blot.

### 2.9. Determination of IL-6, IL-10, TNF-*α*, and VEGF Levels

According to the manufacturer's instructions, the concentrations IL-6, IL-10, TNF-*α*, and VEGF were quantified by enzyme-linked immunosorbent assay. IL-6, IL-10, TNF-*α*, and VEGF kits were obtained from R&D Systems (R&D Systems, CA).

### 2.10. Transfection

An RNA-guided CRISPR/Cas9-mediated genome editing approach was used to delete Nr1d1 gene in BGC-823 cells. Rev-erb*α* targeting sgRNAs were purchased from Santa Cruz (Cat#: SC-401211), which was cloned into a lentiCRISPRv1 plasmid (Addgene, Cambridge, MA). This generated lentiCRISPR-Rev-erb*α*-sgRNA vector. A lentiCRISPRv1 plasmid which expressed an EGFP targeting sgRNA (Addgene) only was used to generate control lentiCRISPR-EGFP-sgRNA vector [21]. Cells were seeded in a 6-well cell culture plate at 2 × 10^5^ cells/well in medium and transfected with lentiCRISPR-Rev-erb*α*-sgRNA vector or control lentiCRISPR-EGFP-sgRNA vector for 48 h [21]. Cells were then selected in medium containing puromycin (1 *μ*g/ml) for 2 weeks. Nr1d1 gene expression was determined by qRT-PCR.

### 2.11. Chromatin Immunoprecipitation (ChIP) Assay

Cells were fixed with 1% formaldehyde and terminated with 2.5 mM glycine. The scraped cells were sonicated for lysis in PBS with sodium thiosulfate. The lysates were divided into three aliquots, one of which was a positive control and received no treatment and one of which was a negative control, which was incubated with target protein. One-third of the cell lysate was served as the test group and was incubated with antibody against Rev-erb*α* (1 *μ*g) and Protein G PLUS-Agarose. After removal of RNA and protein, DNA was extracted with phenol-chloroform, respectively. Next, the degree of enrichment on gene promoters was detected using real-time quantitative PCR.

### 2.12. Statistical Analysis

SPSS 20.0 software was using for statistical analysis. The results were subjected to one-way ANOVA and parametric t-testing and were expressed as the mean ± SEM. Statistical significance was accepted at a level of *P* value <0.05.

## 3. Results

### 3.1. MNU/*H. pylori* Induces Gastric Tumors in Mice

Gastric tumors were examined macroscopically and microscopically by a pathologist in a blind manner. Overall, Table 2 shows that 29.2% of mice developed gastric tumors after MNU/*H. pylori* treatment for 6 months, whereas 86.4% of mice developed gastric tumors after being fed MNU/*H. pylori* for 12 months. These results demonstrate that MNU/*H. pylori* induced gastric cancer in mice.

### 3.2. Levels of Rev-Erb*α* Protein Are Specifically Decreased in Gastric Mucosal Epithelial Cells in Clinical Gastric Cancer Tissues

**Figure 1:**
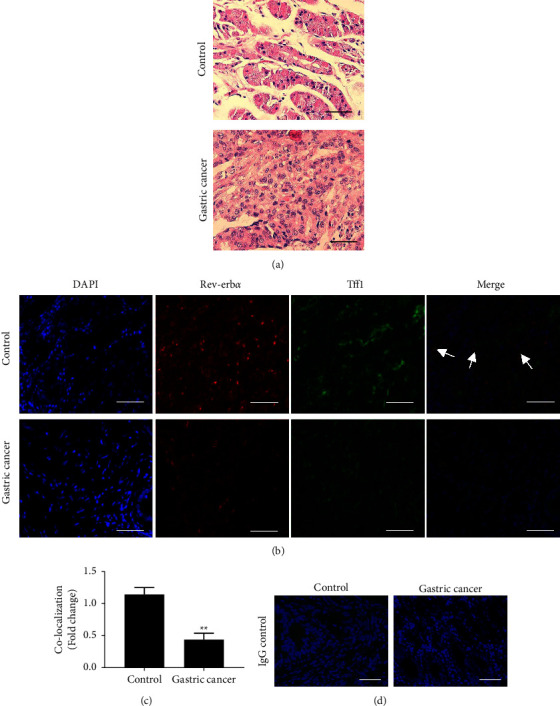
Rev-erb*α* protein is specifically decreased human gastric cancer tissues. Human gastric cancer tissues (gastric cancer) and normal tissues adjacent to cancer (control) were used. (a) H&E staining was performed in gastric tissues with 400x magnification, and representative images are shown. (b) Immunofluorescent double-staining for Rev-erb*α* and a gastric mucosal epithelial cell marker Tff1 was performed with 200x magnification. Representative images shown are those that were captured by a fluorescence microscope. Arrows denote co-localization of Rev-erb*α* and Tff1. (c) Co-localization signals of Rev-erb*α* and Tff1 were analyzed. (d) IgG control staining was performed in gastric cancer tissues and adjacent normal tissues. Scale bar: 50 *μ*m. Mean ± SEM, *N * = 6. ^*∗∗*^*P* < 0.01 vs the control group.

To determine whether Rev-erb*α* was decreased in human gastric cancer tissues, Rev-erb*α* and a gastric mucosal epithelial cell specific marker (Tff1) were detected in human gastric cancer tissues and the corresponding adjacent tissues by immunofluorescence staining. First, we confirmed that gastric cancer tissues with moderately differentiated adenocarcinoma through H&E staining (Figure 1). Compared with the corresponding adjacent tissues, the fluorescence intensity of Rev-erb*α* protein in human gastric cancer tissues was significantly lower. The fluorescence intensity of Rev-erb*α* protein was also reduced in Tff1-positive cells determined by co-localization analysis (Figures 1(b) and 1(c)), while IgG control showed no signals (Figure 1(d)). These results suggest that Rev-erb*α* protein is specifically reduced in gastric mucosal epithelial cells in clinical human gastric cancer tissues.

### 3.3. Levels of Rev-Erb*α* Are Decreased in Gastric Tissues, and Lactic Acid Levels Are Increased in Mice with MNU/*H. pylori*-Induced Gastric Cancer and in Human Gastric Cancer Tissues

**Figure 2:**
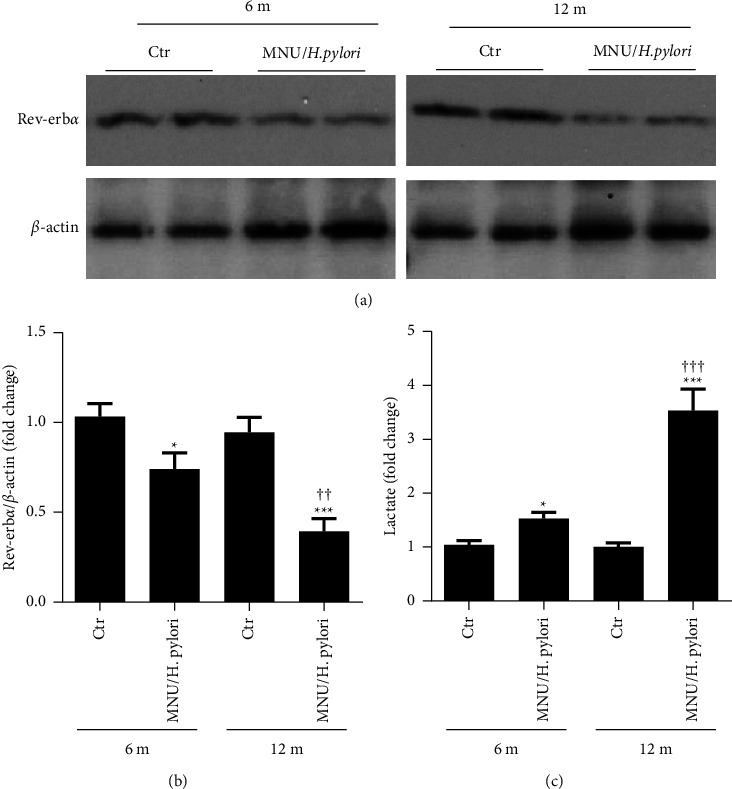
MNU/*H. pylori* decreases the levels of Rev-erb*α* in gastric cancer tissues of mice accompanied by an increase in the level of lactic acid. After C57BL/6J mice were exposed to MNU (200 ppm) for 10 weeks, they were inoculated with *H. pylori* (5 × 10^9^ CFU/time, 5 times in total). After 6 or 12 months (m) of MNU exposure, the levels of Rev-erb*α* protein (a), (b), and lactic acid (c) in the gastric tissues of mice were measured. Mean ± SEM, *N * = 8. ^*∗*^*P* < 0.05, ^*∗∗∗*^*P* < 0.001 vs corresponding controls (Ctr); ^††^*P* < 0.01, ^†††^*P* < 0.001 vs MNU/*H. pylori* (6 m).

To determine whether the formation of gastric tumors induced by MNU/*H. pylori* was associated with Rev-erb*α*, we measured Rev-erb*α* protein levels in experimental gastric tissues. Compared with the control group, significantly reduced levels of the Rev-erb*α* protein were observed in the gastric tissues of the MNU/*H. pylori* groups in a time-dependent manner (Figures 2(a) and 2(b)). Furthermore, the levels of lactic acid in the stomach tissues of the MNU/*H. pylori* treatment group were significantly higher than those in the control group (Figure 2(c)). Additionally, lactic acid was increased in human gastric cancer tissues compared to that in adjacent normal gastric tissues (Table 1). These results suggest that the formation of gastric tumors is associated with a decrease in Rev-erb*α* levels and an increase in glycolysis.

### 3.4. Rev-Erb*α* Phosphorylation Levels Are Unchanged in BGC-823 Cell Lines or in MNU/*H. pylori*-Induced Moues Gastric Tumor Tissues

**Figure 3:**
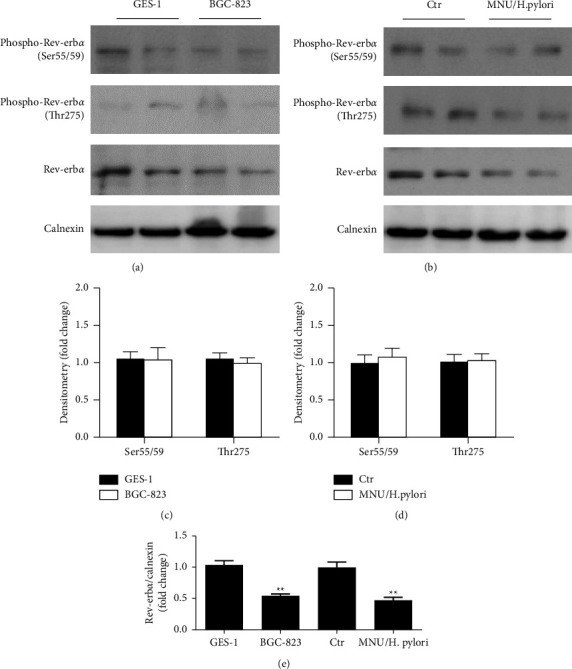
Rev-erb*α* protein phosphorylation is unchanged in gastric cancer cells line BGC-823 and in MNU/*H. pylori*-induced mouse gastric tumor tissues. (a, c) Levels of Rev-erb*α* protein phosphorylation were determined in normal human gastric mucosal epithelial cells (GES-1) and human gastric cancer cell lines (BGC-823) in the logarithmic growth phase by Western blot. (b, d) Rev-erb*α* protein phosphorylation at Ser55/59 and Thr275 was detected in normal control mice and MNU/*H. pylori*-induced mouse gastric cancer tissues. Calnexin was used as a housekeeping control. Mean ± SEM, *N * = 4–6.

We examined changes in Rev-erb*α* protein phosphorylation in human gastric cancer cell line (BGC-823) and in gastric cancer tissues from MNU/*H. pylori*-exposed mice to determine whether it is associated with the decreased expression of Rev-erb*α* protein. Compared with normal human gastric mucosal epithelial cells (GES-1), there was no significant change in phosphorylation on the Ser55/59 or Thr275 amino acid residues of Rev-erb*α* protein in BGC-823 (Figures 3(a) and 3(c)). Similarly, in MNU/*H. pylori*-induced mouse gastric cancer tissues, phosphorylation on Ser55/59 or Thr275 amino acid residues of Rev-erb*α* protein were not significantly changed compared with controls (Figures 3(b) and 3(d)). These results suggest that Rev-erb*α* phosphorylation levels is unchanged in BGC-823 cell lines or in MNU/*H. pylori*-induced moues gastric cancer tissues.

### 3.5. SUMO Modification of Rev-Erb*α* Is Significantly Increased in BGC-823 and MNU/*H. pylori*-Induced Mouse Gastric Cancer Tissues

**Figure 4:**
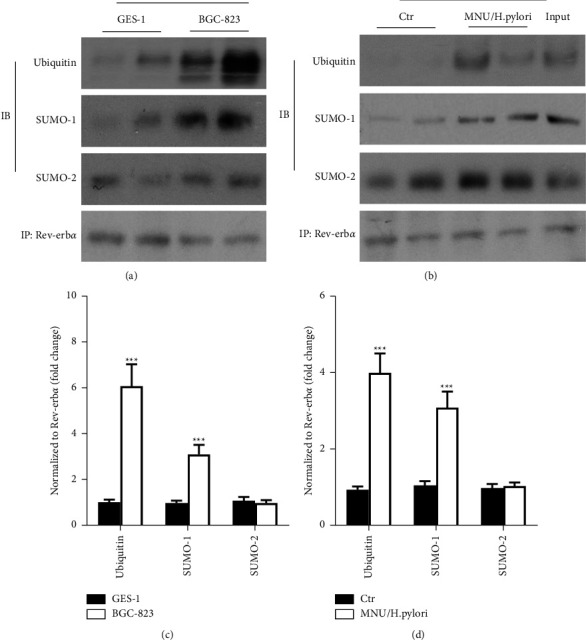
Rev-erb*α* protein SUMOylation and ubiquitination modifications are significantly increased in gastric cancer cells line BGC-823 and in MNU/*H. pylori*-induced moues gastric tumor tissues. (a, b) Rev-erb*α* protein SUMOylation and ubiquitination modifications in normal human gastric mucosal epithelial cells (GES-1), human gastric cancer cell lines (BGC-823) (a), control mouse gastric tissues (Ctr), and MNU/*H. pylori*-induced mice gastric cancer tissues (b) were detected by immunoprecipitation. (c, d) Ubiquitin, SUMO1, and SUMO2 were standardized with the corresponding Rev-erb*α*. Mean ± SEM, *N * = 4–5. ^*∗∗∗*^*P* < 0.001 vs GES-1 or Ctr. IP: immunoprecipitation; IB: immunoblot.

The levels of Rev-erb*α* SUMOylation in BGC-823 cell lines and in MNU/H. pylori-induced moues gastric cancer tissues were detected by immunoprecipitation. As shown in Figures 4(a) and 4(c), compared with normal human gastric mucosal epithelial cells (GES-1), interactions between Rev-erb*α* protein and ubiquitin were significantly increased in the BGC-823 cell line. Furthermore, interactions between Rev-erb*α* protein and SUMO1 were increased in the BGC-823 cell line compared to GES-1. Similarly, both ubiquitination and SUMOylation of Rev-erb*α* were increased in MNU/*H. pylori*-exposed mouse gastric tumor tissues (Figures 4(b) and 4(d)). However, the interaction between Rev-erb*α* protein and SUMO2 was unchanged in the BGC-823 cell line or in MNU/*H. pylori*-induced mouse gastric cancer tissues. These data suggest that Rev-erb*α* SUMOylation and subsequent ubiquitination may contribute to its degradation in gastric cancer tissues.

### 3.6. IL-6 IL-10, TNF-*α*, and VEGF Are Increased in BGC-823 Cell Lines, Serum of MNU/*H. pylori*-Exposed Mice, and Human Gastric Cancer Tissues

**Figure 5:**
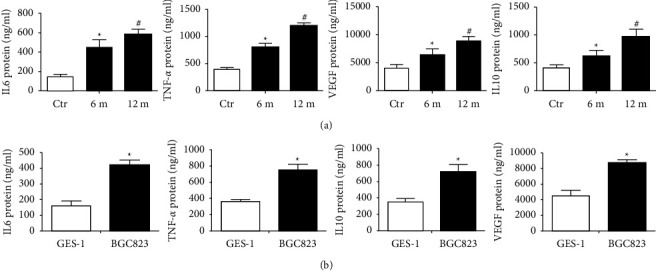
IL-6, IL-10, TNF-*α*, and VEGF levels are increased in serum from MNU/*H. pylori*-induced mice and in supernatants from BCG-832 cell lines. IL-6, IL-10, TNF-*α*, and VEGF protein concentrations in serum of experimental mice (a) and in supernatants from GES-1 and BCG-832 cells were measured by ELISA. Mean ± SEM, *N * = 4-5. ^*∗*^*P* < 0.05 vs control group. ^#^*P* < 0.05 compared with 6-month group. 6 m: 6-month; 12 m: 12-month.

We quantified IL-6 and TNF-*α* levels in serum samples from mice to determine any significant differences in cytokine concentrations between the normal and experimental groups. The method was also used to compare GES-1 cells and the BGC-823 cell line. We found that a significant increase in IL-6 and TNF-*α* concentration was observed in serum samples from MNU/*H. pylori*-exposed mice compared to control mice (Figure 5(a)). Levels of IL-6, IL-10, TNF-*α*, and VEGF were higher in the BGC-823 cell lines compared with those of GES-1 cells (Figure 5(b)). Likewise, the levels of these cytokines were increased in gastric cancer tissues compared to those in adjacent normal tissues (Table 1). These results suggest that inflammatory responses are increased in gastric cancer, which is associated with reduced Rev-erb*α*.

### 3.7. IL-6 and IL-1 Treatments Do Not Affect Rev-Erb*α* Protein but Increase Lactic Acid Levels in Cultured Human Gastric Cancer Cells

**Figure 6:**
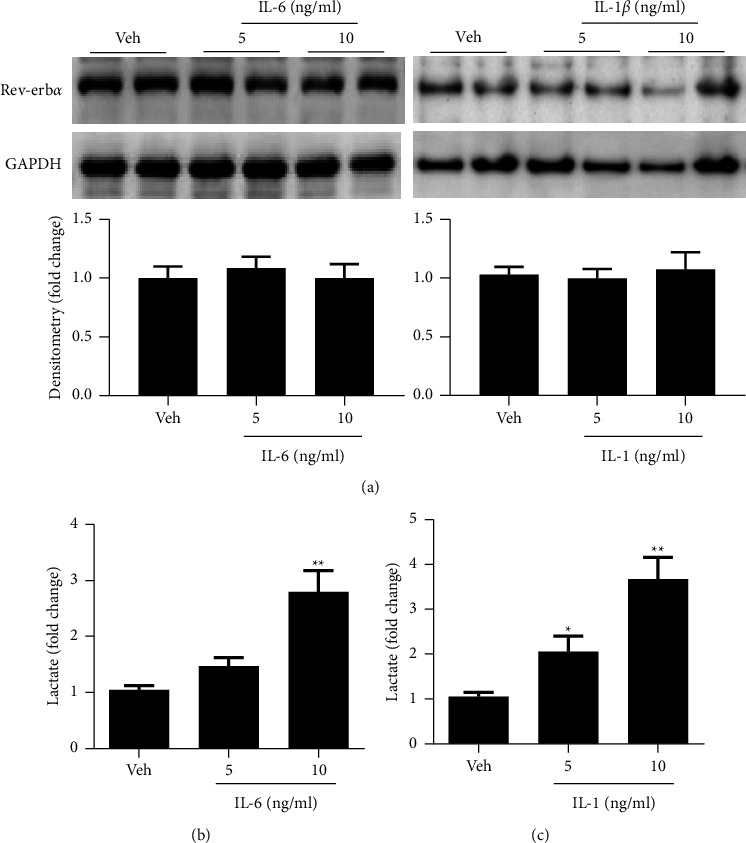
IL-6 and IL-1 treatments do not affect Rev-erb*α* protein levels but increase lactic acid levels in cultured human gastric cancer cells. BGC-823 cells were treated with IL-6 (5 and 10 ng/ml) or IL-1 (5 and 10 ng/ml) for 24 hours. (a) Rev-erb*α* levels were measured by Western blot. (b) Lactic acid was measured using a kit. Mean ± SEM, *N * = 4-5.

BGC-823 cells were treated with IL-6 or IL-1 (5 and 10 ng/ml) for 24 h. Rev-erb*α* levels were measured by Western blot. As shown in Figure 6, IL-6 and IL-1 treatments did not affect Rev-erb*α* protein levels (Figure 6(a)). Interestingly, the levels of lactic acid were significantly increased in these cells treated with IL-6 and IL-1 (Figures 6(b) and 6(c)). This suggests that inflammation may not cause Rev-erb*α* reduction but increases glycolysis in human gastric cancer cells.

### 3.8. Rev-Erb*α* Is Recruited on the Promoter of Cytokine Genes, Which Represses Their Expression

**Figure 7:**
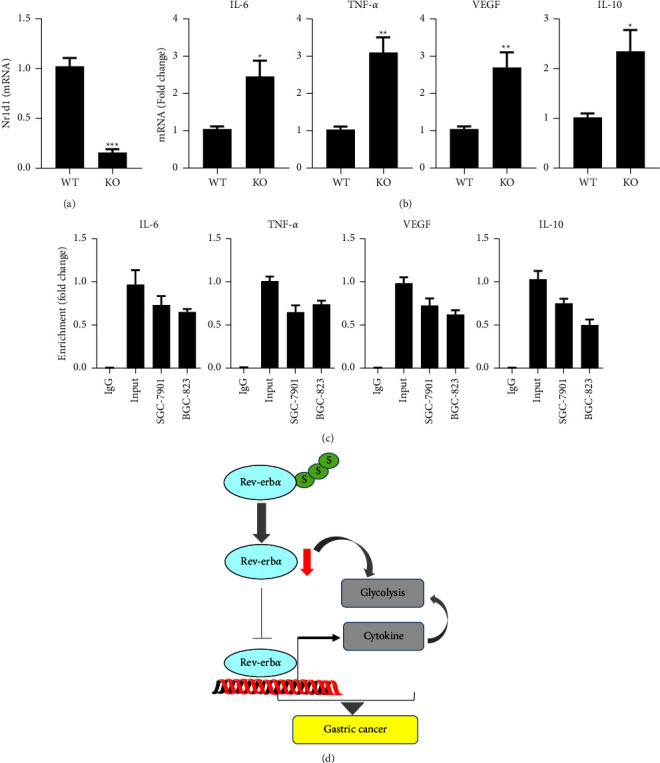
Rev-erb*α* is recruited on the promoters of cytokines, and Nr1d1 gene deletion increases expression of these cytokine genes. (a, b) The expression of Nr1d1, IL-6, TNF-*α*, VEGF, and IL-10 genes was evaluated by qRT-PCR in Nr1d1 knockout and WT BGC-823 cells. (c) ChIP was performed to detect the recruitment of Rev-erb*α* on the promoters of IL-6, TNF-*α*, VEGF, and IL-10 genes in both BGC-823 and SGC-7901 cells. (d) Schematic figure showing that Rev-erb*α* SUMOylation may cause its protein degradation, and this decreases its recruitment on promoters of cytokine genes, leading to increased glycolysis. Mean ± SEM, *N * = 4-5. ^*∗*^*P* < 0.05, ^*∗∗*^*P* < 0.01 vs WT group.

Since IL-1 and IL-6 did not affect Rev-erb*α* protein levels, we wanted to evaluate whether Rev-erb*α* inhibits cytokine gene expression. First, we deleted Rev-erb*α* encoding gene Nr1d1 and determined expression of cytokine genes. As shown in Figures 7(a) and 7(b), Nr1d1 gene expression was significantly reduced in Nr1d1 KO cells. Nr1d1 gene deletion remarkedly increased the expression of IL-6, TNF-*α*, VEGF, and IL-10 levels in BGC-823 cells. Furthermore, Rev-erb*α* protein was recruited on the promoter of IL-6, TNF-*α*, VEGF, and IL-10 genes (Figure 7(c)). These results suggest that Rev-erb*α* is recruited on the promoter of cytokine genes, which represses their expression.

## 4. Discussion

Rev-erb is a nuclear receptor and transcriptional repressor, and the two family members Rev-erb*α* and Rev-erb*β* are encoded by the Nr1d1 and Nr1d2 genes, respectively. Rev-erbs participate in pathological processes including sleep disorders, diabetes, fatty liver, atherosclerosis, Alzheimer's disease, and abnormal bone resorption/remodeling by regulating the biological clock, inflammatory/immune responses, and lipid metabolism [22–24]. Data from a study by Sulli et al. showed that Rev-erb agonists reduced the survival of brain cancer, leukemia, breast cancer, rectal cancer, and melanoma cell lines [10]. Additionally, the activation of Rev-erb*α*/*β* inhibited the growth of mouse glioblastoma [10, 11]. We recently reported that Rev-erb*α* is reduced in human gastric cancer tissues with an increased TMN stage. Furthermore, the low expression of Rev-erb*α* is associated with poor prognosis in gastric cancer patients [13]. Rev-erb*α* was also reduced in MNU/*H. pylori*-induced gastric cancer tissues. Specifically, Rev-erb*α* was decreased in gastric mucosal epithelial cells in gastric tissues, suggesting epithelial cell differentiation and tumorigenesis. The role of Rev-erb*α* in the development of gastric cancer will be demonstrated using KO mice in future. Further study is warranted to determine the mechanisms of reduced Nr1d1 gene expression in gastric cancer [12, 13].

Rev-erb*α* can undergo various protein modifications through ubiquitination/proteasome-dependent degradation pathways that affect its stability. For example, phosphorylation of serine (Ser) residues 55 and 59 of Rev-erb*α* protein increased its stability, whereas phosphorylation of threonine residue (Thr) 275 reduced its stability [25, 26]. Additionally, phosphorylation of N-terminal regions of Rev-erb*α* regulates its intracellular localization and signal pathway [26, 27]. On the basis of these findings, we tested whether Rev-erb*α* was phosphorylated in human gastric cancer cell lines and MNU/*H. pylori*-induced mouse gastric cancer tissues. There were no significant changes in the levels of Rev-erb*α* protein phosphorylation (Ser55/59 and Thr275) in human gastric cancer cell lines and in mouse gastric cancer induced by MNU/*H. pylori*, suggesting the decrease of Rev-erb*α* protein in gastric cancer tissues may not be related to its phosphorylation. Despite this, the ubiquitination of Rev-erb*α* protein was significantly increased in human gastric cancer cell lines and mouse gastric cancer tissues. These results suggest that the Rev-erb*α* protein might undergo other modifications in gastric cancer tissues that cause it to bind to ubiquitin, which leads to its proteasome-dependent degradation.

In HEK293 cells, Rev-erb*α* protein can undergo SUMO modification under the stimulation of inflammatory factors, leading to its ubiquitination and proteasome-dependent degradation [28]. SUMO is a ubiquitin-like protein with four family members: SUMO1, SUMO2, SUMO3, and SUMO4. SUMO1-SUMO3 are expressed in all tissues, whereas SUMO4 is expressed specifically in organs. The SUMO modification covalently binds SUMO to the amino acid residues of the target protein by activating enzyme E1, combining enzyme E2 (Ubc9) and ligase E3. It is a dynamic and reversible process, and deSUMOylation is mediated by SUMO-specific protease family members. We, for the first time, found that SUMO modification of Rev-erb*α* protein was significantly increased in human gastric cancer cell lines and mouse gastric cancer tissues. This may be related to the marked increase in SUMO1 expression in human gastric cancer tissues [29]. Further studies using proteasome inhibitors, ubc9, and SUMO1 transfection in gastric cancer cells would further understand whether Rev-erb*α* SUMOylation causes its protein degradation.

Current research of Rev-erbs in metabolism has mainly focused on lipid metabolism. For example, when the Rev-erb*α* gene is knocked out in mice, the expression of apolipoprotein CIII in the liver and serum is increased, and the levels of very low-density lipoprotein and triglycerides are significantly increased [30]. Additionally, the expressions of key genes involved in fatty acid metabolism (CD36, Fabp3, and Fabp4) were decreased in cells containing Rev-erbs mutants [31]. In terms of glucose metabolism, Rev-erb*α* inhibits gluconeogenesis. When heme binds to Rev-erb*α* in hepatocytes, it enhances its activity and inhibits the gene expression of a key enzyme (phosphoenolpyruvate carboxykinase, PEPCK) required for gluconeogenesis [32]. We reported that Rev-erb*α* reduction causes gastric cancer cell proliferation by upregulating glycolysis and pentose phosphate pathway (PPP) [20]. The lactate was increased in serum of MNU/*H. pylori*-exposed mice, which may be due to reduced Rev-erb*α*. *H. pylori* has the ability to utilize glucose for metabolism through a glucokinase activity and enzymes of the PPP and glycolysis pathways [33, 34]. Interestingly, the anti-*H. pylori* activity was observed when treated with high levels of exogenous lactate [35]. Thus, it is possible that *H. pylori* increases lactate production for gastric cancer cell proliferation but in return reduces its bacterial activity. Whether Rev-erb*α* inhibits the proliferation of gastric cancer cells in vivo and the growth of gastric cancer remains unclear.

Inflammatory responses play key roles in cancer development, including tumor occurrence, promotion, progression, and metastasis. Cytokines are considered to be important mediators linking inflammation to gastric cancer [36]. Our data suggested significantly increased levels of IL-6, IL-10, TNF-*α*, and VEGF in serum samples from the experimental mice compared with normal mice. Furthermore, the levels of IL-6, IL-10, TNF-*α*, and VEGF in the gastric cancer cell line were higher than those in normal human gastric mucosal epithelial cells. Research studies have showed that the decreased activity of Rev-erb*α* or Rev-erb*α* knockout promoted the production of IL-6 and TNF-*α* in rodent lungs [6, 7]. This is corroborated by our findings showing increased expression of these genes in Nr1d1 KO cells. These findings suggest that increased inflammatory response is associated with reduced Rev-erb*α* during the development of gastric cancer. It is noted that *H. pylori* induces inflammation [37, 38]. Further studies are required to dissect the role of *H. pylori* and reduced Rev-erb*α* in modulating inflammatory responses in gastric cancer.

IL-1 causes Rev-erb*α* SUMOylation, leading to its degradation in HEK293T cells [28]. Interestingly, IL-6 and IL-1 incubation did not affect Rev-erb*α* protein levels in gastric cancer cells. Cytokines could stimulate glycolysis to promote cancer cell proliferation [39–41]. This is the case in our findings showing increased lactate by IL-1 and IL-6 incubation. We speculate that increased concentrations of cytokines may promote glycolysis pathway in patients with gastric cancer.

## 5. Conclusion

In summary, this is the first study to show that the SUMO modification of Rev-erb*α* protein is observed in gastric cancer tissues, which is associated with protein degradation. Rev-erb*α* reduction causes the expression of cytokine genes due to reduced recruitment on their promoters. Increased release of cytokines augments glycolysis, which is seen in gastric cancer (Figure 7(d)). Targeting Rev-erb*α* or its SUMO modification may represent promising approaches for prevention and management of gastric cancer.

## Figures and Tables

**Table 1:** Characteristics of human subjects with gastric cancer.

Subject	Age (years)	Gender	*IL-6 (pg/mg protein)*		*TNF-α (pg/mg protein)*		*Lactic acid (mmol/mg protein)*	
			Adjacent normal	Tumor tissues	Adjacent normal	Tumor tissues	Adjacent normal	Tumor tissues
1	55	Male	20.4	100.2	254.1	452.2	4.25	9.85
2	63	Male	35.4	195.2	169.2	742.1	5.81	15.84
3	65	Female	41.2	174.2	198.5	642.1	6.24	13.2
4	58	Male	56.8	303.6	208.2	841.2	4.15	18.4
5	58	Male	54.1	250.2	175.2	623.7	3.51	12.8
6	60	Male	62.4	274.4	169.8	584.1	2.84	11.2

**Table 2:** Incidence and multiplicity of MNU/*H. pylori*-induced gastric tumor.

Group	Total mice	Dead mice	Tumor-bearing mice, *n* (%)	Gastric adenoma, *n* (%)	Gastric adenocarcinoma, *n* (%)
Control	10	0	0	0	0
MNU/*H. pylori* (6 m)	25	1	7 (29.2%)	6 (25%)	2 (8%)
MNU/*H. pylori* (12 m)	25	3	19 (86.4%)	8 (33.3%)	15 (68.2%)

## Data Availability

The data used to support the findings of our present research are available from the corresponding authors upon request.

## Additional Points

Contribution to the field. Although gastric cancer is a common malignant tumor in humans, its pathological mechanism is poorly understood. We reported that Rev-erb*α* protein is decreased in human gastric cancer, which is associated with poor differentiation, TMN stages, and poor prognosis. However, it is unclear whether Rev-erb*α* protein undergoes posttranslational modifications leading to its degradation in gastric cancer. Here, we found that Rev-erb*α* SUMOylation and subsequent ubiquitination were increased in cultured gastric cancer cells and in N-methyl-N-nitrosourea/*Helicobacter pylori*-induced mouse gastric tumor tissues. This was associated with increased glycolysis and abnormal inflammatory responses. Therefore, targeting Rev-erb*α* and blocking SUMOylation-mediated protein degradation represent a promising approach for prevention and management of gastric cancer.
